# Non-Invasive Saliva-based Detection of Gene Mutations in Oral Cancer Patients by Oral Rub and Rinse Technique

**DOI:** 10.31557/APJCP.2021.22.10.3287

**Published:** 2021-10

**Authors:** Audrey D’Cruz, Pandyanda Nanjappa Dechamma, Marina Saldanha, Sahana Maben, Pushparaja Shetty, Anirban Chakraborty

**Affiliations:** 1 *Nitte (Deemed to be University), AB Shetty Memorial Institute of Dental Sciences, Department of Public Health Dentistry, Mangaluru, Karnataka, India. *; 2 *Nitte (Deemed to be University), Nitte University Centre for Science Education and Research (NUCSER), Division of Molecular Genetics and Cancer, Mangaluru, Karnataka, India. *; 3 *Nitte (Deemed to be University), KS Hegde Medical Academy, Department of Otorhinolaryngology, Mangaluru, Karnataka, India. *

**Keywords:** Tobacco, Cessation counseling, Tobacco quitting, Quitting perception, India

## Abstract

**Background:**

Oral Squamous Cell Carcinoma (OSCC) is the most widely reported cancer worldwide. Liquid biopsy, a method that relies on identification of tumor-associated cells and/or cell free nucleic acids from body fluids is becoming increasingly popular in cancer diagnostics. The aim of the study was to evaluate the feasibility of Oral Rub and Rinse (ORR) technique in determining the genetic changes in common biomarkers of oral cancer such as TP53 using DNA obtained from saliva of oral cancer patients.

**Methods:**

A total of 15 oral cancer patients were recruited in the study and pre-surgical saliva samples were collected using the ORR technique. Tissue samples included in the study were obtained during the surgical excision of the cancerous oral lesion. Genomic DNA was isolated from the salivary cell plug and the tissues and the *TP53* gene was amplified by PCR. The PCR products of all the exons of TP53 (Exons 2 to 11) were electrophoresed on agarose gel, purified and sequenced by Sanger method. The obtained sequences were compared with the reference sequence of *TP53* gene.

**Statistical analysis used:**

Descriptive statistics were used and reported as frequency and percentage.

**Results:**

Capillary sequencing of TP53 gene from tissue DNA revealed the presence of codon 72 c.215C>G (p.Pro72Arg) polymorphism in 10 patients (67%) and a heterozygous mutation at codon 172 c.514 G>T (p.Val172Phe) in 2 patients (13%). Among the 10 samples that showed codon 72 polymorphism, matched salivary DNA was available for 6 samples and 4 out of these showed same genetic change at codon 72. Similarly, of the 3 samples that showed codon 172 mutation, matched salivary DNA was available for 1 sample and the mutation status was identical.

**Conclusion:**

The results suggest a potential for clinical applications of ORR technique as an alternative to invasive tissue biopsy for detection of genetic changes in candidate biomarkers in oral cancer.

## Introduction

Cancer of the oral cavity is one of the common malignancies around the world. As per Globocan 2020 data, 3.7 million new oral cancer cases were reported worldwide and a staggering 64.2% were from Asia (Global Cancer Observatory, 2020). Oral cancer was the second most common type in incidence and the third most common type in cancer-associated mortality (Global Cancer Observatory, 2020). Oral Squamous cell carcinoma (OSCC) is the most prominent subtype of oral cancer accounting for nearly 90% of the cases (Shah and Gill, 2009). In India, OSCC is the most common cancer among males and it ranks third among females (Salian et al., 2016). Despite advancements in therapeutic strategies, the incidence of OSCC and the associated mortality is high due to its poor diagnosis. Early diagnosis of oral cancer can play a pivotal role in instituting early treatment and thus improving the prognosis. Biopsy and histopathology have been the ‘gold standards’ for diagnosis of OSCC. In recent years, considerable attention has been paid on developing simple, cost effective and non-invasive methods for early diagnosis of OSCC.

Tumor biomarkers are substances that are produced either by the tumor cells or by the host immune mechanisms, which are released in the body fluids. Monitoring these biomarkers in body fluids can help in the early detection and diagnosis of cancer. Indeed, several tumor markers have been identified for OSCC (Negi et al., 2018; Economopoulou et al., 2019). Molecular identification of such tumor markers in early stage of OSCC would permit optimal timing for treatments and consequently prolonged survival. Saliva, a store house of various enzymes, nucleic acids and proteins is a promising, non-invasive and easy source of tumor biomarkers for diagnosis and prognosis of OSCC. Liquid biopsy, a method that relies on identification of tumor-associated cells and/or cell free nucleic acids from body fluids is becoming increasingly popular in cancer diagnostics. Tumour-derived exosomal (TEX) biomarkers obtained from a liquid biopsy is a promising tool in OSCC diagnosis (Sahu and Routray, 2021).

TP53, a tumor suppressor protein that plays an important role in the control of cell division, is one of the important biomarkers of oral cancer. Several reports have shown the possible association of *TP53* gene polymorphism in oral, breast, bladder, colorectal and lung cancers (Soulitzis et al., 2002; Katiyar et al., 2003; Zhu et al., 2007; Rivlin et al., 2011; Hanel and Moll 2012). TP53 mutations have shown to be associated with an increased risk of extranodal extensions in patients with advanced OSCC (Gleber-Netto et al., 2020). According to the IARC database (2018), 42.51% of head and neck cancers carry somatic mutations in *TP53*. Studies have reported single nucleotide polymorphism (SNP) at the* TP53 *exon 4 codon 72, as a risk factor for development of cancer including OSCC (Sand et al., 2012).

Previous studies have shown that the Oral Rub and Rinse (ORR) technique is better than conventional exfoliative cytology methods in terms of cellular clarity and preparation of multiple smears (Mulki et al., 2013). However, studies on its feasibility in detection of genetic alterations have been limited. In this study, the aim was to evaluate the feasibility of ORR technique in determining the genetic changes in common biomarkers of oral cancer such as TP53 using DNA obtained from saliva of clinically confirmed oral cancer patients. 

## Materials and Methods

*Selection of study participants*

A total of 15 patients were included in the present study. Subjects above 18 years who were diagnosed clinically and histologically with Oral Squamous Cell Carcinoma (OSCC) and willing to participate in the study were included. Patients with previous history of malignancy and those that underwent cancer treatments (surgery, chemotherapy, radiotherapy) were excluded from the study. Also, subjects with any prior history of exposure to radiation, carcinogens or heavy metals were excluded from the study. A proforma was used to collect the socio-demographic details along with the history of tobacco use. 

*Sample collection and processing*

Of the 15 patients, pre-surgical saliva samples were collected from 6 patients using the Oral Rub and Rinse technique. In ORR technique, each patient was asked to rinse mouth with water to clear any debris. Post rinsing, the lesion was rubbed for 30 seconds by the clinician’s gloved finger with firm finger pressure. The patient was then asked to swish the mouth with 10 mL of 1% phosphate buffered saline (pH7.2) and expectorate into a sterile, labelled container. The collected saline solution was centrifuged at 1,000 rpm for 5 minutes to obtain a cell plug. After discarding the supernatant fluid, the cell plug was pipetted and stored at -20ºC till further processing. 

Tissue samples included in the study were obtained from 15 patients during the surgical excision of the cancerous oral lesion. The tissue samples obtained were stored at -20ºC till further processing. Ethical Clearance for the study was obtained from the Central Ethics Committee of the University (NU/CEC/2020/0297).

*Isolation of genomic DNA and amplification of coding region of TP53*

Genomic DNA were isolated from the salivary cell plug and the tissues using the DNeasy Blood & Tissue Kit (Qiagen, Germany) following manufacturer’s instructions. Using the extracted DNA as template, the *TP53* gene was amplified by PCR in an automated thermal cycler (Eppendorf, Germany) using exon-specific primers. A total of seven PCR reactions were performed for each sample to cover the entire coding region of TP53 (exon 2 to exon 11). The sequences of the primers used for PCR are mentioned in Supplementary Table 1. 

*Capillary sequencing of TP53 gene*

The PCR products of all the exons of *TP53* (Exons 2 to 11) were electrophoresed on agarose gel, purified and sequenced by Sanger method. The sequencing reactions were outsourced to commercial vendors. The presence or absence of polymorphism/variation for each codon was determined by comparing the obtained sequences with the reference sequence of *TP53* gene (NM_000546.6; *NCBI* Gene: 7157; HGNC:11998).

*Statistical analysis*

The data was entered in Ms Excel for Windows. Descriptive statistics were used in the study and reported as frequency and percentage.

## Results

*Socio-demographic details of OSCC patients*

The socio-demographic details with tobacco use history of the study participants are given in Table 1. The mean age of the study population was 54.6 + 9.99years with 73.3 % males and 26.7% females. About 93% of the cases (11 males and 3 females) included in the study had history of smokeless tobacco use (betel quid with tobacco). One female patient did not have any prior history of tobacco usage. 

*TP53 gene mutations*

Sanger sequencing of the coding regions of *TP53* gene in 15 patients revealed the presence of two missense somatic mutations. A homozygous mutation at codon 72 c.215C>G (p.Pro72Arg) was observed in 10 patients (67%). In addition, a heterozygous mutation at codon 172 c.514 G>T (p.Val172Phe) was observed in 2 patients (13%).

**Figure 1:**
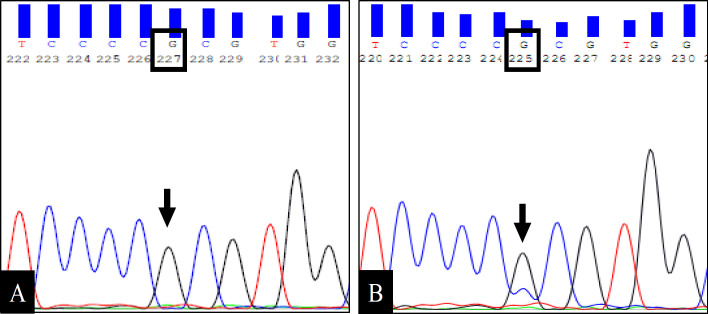
Representative Sequence Data of *TP53 *Gene Amplified from Matched Salivary (A) and Tissue DNA (B) of an OSCC Patient. As shown in A, the patient displays homozygous mutation (C>G) in coding nucleotide position 215 (arrow) resulting in a missense mutation at codon 72 leading to change of amino acid proline (CCC) to arginine (CGC). The same change is seen in the *TP53 *gene amplified from the tissue DNA (B)

To determine whether the *TP53* somatic mutations identified from the tissue samples can be picked up in DNA obtained from salivary cell plug by ORR technique from the same patient, Sanger sequencing of *TP53 *coding region was carried out for all the 6 patients where pre-surgical salivary DNA was available. Of the 6 matched samples (availability of both salivary and tissue DNA), 4 samples showed identical mutation pattern at codon 72 c.215C>G (p.Pro72Arg) of *TP53* gene (Figure 1). 

**Figure 2:**
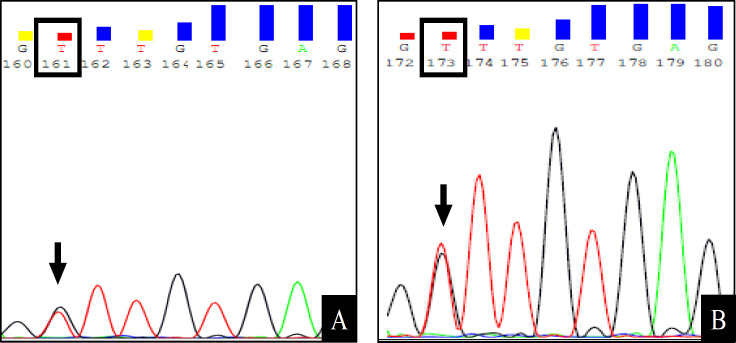
Representative Sequence Data of *TP53 *Gene Amplified from Matched Salivary (A) and Tissue DNA (B) of an OSCC Patient. As shown in A, the patient displays heterozygous mutation (G>T) in coding nucleotide position 514 (arrow) resulting in a missense mutation at codon 172 leading to change of amino acid valine (GTT) to (TTT) in one allele of *TP53* gene. The same change is seen in the *TP53* gene amplified from the tissue DNA (B)

Similarly, among the 2 samples that harboured *TP53 *codon 172 mutation, matched salivary and tissue DNA was available for only one patient. Expectedly, the same mutation was observed in both tissue and salivary DNA (Figure 2). Interestingly, the patient with codon 172 mutation also harboured the codon 72 c.215C>G (p.Pro72Arg) polymorphism.

**Table 1:** Socio-Demographic Details with Tobacco Use History of the Study Participants

Mean age		54.6 + 9.99
Gender	Males	11
	Females	4
Tobacco use	Smokers	0
	Smokeless users	14 (11 male, 3 female)
	Never	01 (Female)
Type of tobacco	Betel quid with tobacco	14
Duration of use	20 years or less	7
	20 – 40 years	6
	50 years or more	1

## Discussion

Cancer is considered as a multi-hit event where a number of genetic aberrations contribute to the malignant transformation. Oral cancer is one of the prevalent cancers in the world and among the various subtypes of oral cancer, squamous cell carcinoma is the most common one (IARC, 2018). 

Biopsy is the first choice for diagnosis of malignant lesions. But conventional biopsy is an invasive procedure which has its own demerits and is practically not possible for terminally ill patients. One of the reasons for poor prognosis in OSCC is late diagnosis of the cancer and it is particularly true in a country like India where most of the cancer cases are diagnosed at stages where the cancer is already advanced. Although conventional oral exfoliative cytology is in practice, there are several disadvantages such as low sensitivity, procedural errors etc (Mulki et al., 2013). Normally shed oral cells can be detected in oral rinses paving a way to cytological investigation assisted by molecular analysis of the fluid for oral cancer screening.

In our study, we used ORR technique for collection of exfoliated epithelial cells, which can serve as a source of cellular DNA for screening of gene mutations. Furthermore, using saliva as a source of DNA, makes it non-invasive, painless, rapid and easy technique to perform.

In this study, the aim was to determine whether exfoliated cancer cells released in saliva in patients with OSCC can be collected by ORR technique and if the DNA extracted from those cells can be used for non-invasive detection of candidate gene mutations. We focused on *TP53* gene, a common biomarker in cancer.TP53 is the most important tumor suppressor gene in a cell, and it is mutated in more than 50% of all cancers (Olivier et al., 2010). We compared the sequence information of *TP53* gene obtained from tissue samples of the patients with those obtained from matched salivary DNA. 

Our data revealed the presence of two mutations in the patients. Sixty seven percent of the patients (10/15) harboured a homozygous mutation at codon 72 c.215C>G (p.Pro72Arg) and the same mutation was observed in 4 out of 6 matched patients where salivary DNA was available. According to the IARC TP53 mutation database (https://p53.iarc.fr/), the codon 72 is one of the validated polymorphisms identified in the *TP53* gene and several studies have been carried out to correlate this polymorphism with cancer risk. However, the results have not been consistent. For instance, Storey et al., (1998) reported that expression of homozygous Arginine 72 increased the susceptibility of cervical cancer up to seven-fold while Liu et al., (2001) showed that the Proline 72 is linked with increased risk for lung carcinoma. Another study by Twu et al., (2006) showed that heterozygous Arginine/Proline genotype is associated with squamous cell carcinoma. However, in our data, all the 12 patients were homozygous for Arg72.

Most mutations in the *TP53* gene occur as point mutations in highly conserved region, i.e., between codons 126–306 (exons 5–8) (Mewara et al., 2010). Mutations that are reported outside exons 5–8 are most common in hepatocellular and bladder carcinomas and are less common in head and neck malignancies. Interestingly, in our data we found that 2/15 OSCC samples (13%) harboured a heterozygous somatic mutation at codon 172 c.514 G>T (p.Val172Phe) and the same was observed from salivary DNA in 1 matched sample. The codon 172 variant is an extremely rare change that has been reported so far. As per ClinVar data, this particular mutation has been observed in a hereditary cancer predisposition syndrome and the clinical significance is likely pathogenic (https://www.ncbi.nlm.nih.gov/clinvar/variation/428909/rs1131691043). Interesingly, the patient with codon 172 mutation also harboured the codon 72 c.215C>G (p.Pro72Arg) polymorphism. The presence of more than one hotspot mutation in a particular gene in the same patient is rare but not uncommon in cancer. For instance, Weng et al, (2019) reported a case of lung cancer, where the patient had both T790M point mutation in exon 20 and L858R point mutation in exon 21 of *EGFR* gene. Our data also highlights the association between tobacco chewing and oral cancer (Wen et al., 2010). We observed that 93.3% of the patients who were tobacco users (betel quid with tobacco) harboured mutation in the *TP53* gene, either in codon 72 or 172, which is in line with previous reports (Chen et al., 2008, Ihsan et al., 2011, Sand et al., 2012). This indicates that patients with TP53 polymorphism are susceptible to development of oral cancer especially in presence of tobacco habit. Interestingly, one female who had no prior history of the use of tobacco also showed the presence of codon 72 polymorphism.

Taken together, the results of our study indicate that the ORR technique can efficiently identify genetic alterations from saliva of oral cancer patients. Of the two identified mutations in the patients, codon 72 polymorphic site was the most common genetic variation seen in 67% of the patients analysed and the ORR technique could accurately identify this change in 66.6% (4/6) of the matched cases. Although the percentage of accuracy was slightly less for this mutation, considering the fact that codon 72 is a validated polymorphic site, the variation of results in remaining 2 matched samples can be accepted. The other mutation, codon 172, which is a rare and is considered a likely pathogenic type, was observed in 13% of the cases and the same was identified by ORR technique in 100% of the matched cases. 

Although our dataset was small, the results strongly indicate that ORR technique can be considered as a suitable alternative to invasive tissue biopsy for detection of genetic changes in candidate biomarkers in oral cancer. Further screening with large number of matched tissue and salivary DNA samples will be necessary to validate the true potential of ORR technique for clinical applications, particularly in molecular diagnostics.

## Author Contribution Statement

Audrey D’Cruz: Conceptualised the study, collected the clinical samples, carried out clinical analysis, obtained funds and prepared the original draft. PN Dechamma: Carried out molecular experiments and involved in data analysis. Marina Saldanha: Collected clinical samples and helped in clinical data curation and compilation. Sahana Maben: Collected clinical samples and helped in clinical data curation and compilation. Pushparaja Shetty: Assisted in study design, participated in discussion and assisted in clinical analysis, reviewed the first draft. Anirban Chakraborty: Supervised the work, analysed the data and edited the draft manuscript and prepared the final manuscript.
